# SENP2 Reduces Hepatocellular Carcinoma Stemness and Improves Sorafenib Sensitivity Through Inactivating the AKT/GSK3β/CTNNB1 Pathway

**DOI:** 10.3389/fonc.2021.773045

**Published:** 2021-12-07

**Authors:** Xiaohui Tang, Bohao Liu, Chen Zhang, Wenbin Tang, Shitian Liang, Yadan Xiao, Ruoyu Deng, Zhuan Li

**Affiliations:** ^1^ Department of Pharmacy, Hunan Normal University School of Medicine, Changsha, China; ^2^ Department of Bioinformatics, School of Life Science, Fudan University, Shanghai, China; ^3^ The Key Laboratory of Model Animals and Stem Cell Biology in Hunan Province, Hunan Normal University School of Medicine, Changsha, China; ^4^ Key Laboratory of Study and Discovery of Small Targeted Molecules of Hunan Province, Hunan Normal University School of Medicine, Changsha, China

**Keywords:** SENP2, hepatocellular carcinoma, stemness, sorafenib sensitivity, AKT/GSK3β/CTNNB1 pathway

## Abstract

**Background:**

Small ubiquitin-like modifier specific peptidase 2 (SENP2) suppresses the progression and chemoresistance of several cancers, while few studies report its role in hepatocellular carcinoma (HCC). This study aimed to evaluate the effect of SENP2 on stemness, sorafenib sensitivity, and downstream pathway in HCC, with validation of its molecular mechanisms by compensation experiment.

**Methods:**

SENP2 was regulated by plasmid transfection; meanwhile, in a compensation experiment, protein kinase B (AKT) was activated by SC79 treatment and β-catenin (CTNNB1) was overexpressed by plasmid transfection. After modification, sorafenib sensitivity was detected by cell counting kit-8 assay; stemness was evaluated by CD133^+^ cell proportion and sphere formation assay.

**Results:**

SENP2 was decreased in HCC cell lines (including Hep3B, Li7, and Huh7) compared with normal human liver epithelial cell lines, which was further reduced in HCC stem cells than in normal HCC cells. Subsequently, SENP2 overexpression inhibited CD133^+^ cell proportion, decreased sphere formation ability, promoted sorafenib sensitivity, suppressed AKT and glycogen synthase kinase-3β (GSK3β) phosphorylation, and reduced CTNNB1 expression in Huh7 and Hep3B cells, while SENP2 knockdown showed the reverse effects. The following compensation experiment revealed that activating AKT or overexpressing CTNNB1 promoted CD133^+^ cell proportion and sphere formation ability but suppressed sorafenib sensitivity in Huh7 and Hep3B cells. Moreover, activating AKT or overexpressing CTNNB1 attenuated the effect of SENP2 overexpression on stemness and sorafenib sensitivity in Huh7 and Hep3B cells.

**Conclusion:**

SENP2 suppresses HCC stemness and increases sorafenib sensitivity through inactivating the AKT/GSK3β/CTNNB1 signaling pathway.

## Introduction

Hepatocellular carcinoma (HCC) is a malignancy causing high morbidity and mortality worldwide (1, 2). Due to the prevalence of hepatitis B virus infection, abuse of alcohol, and other factors, HCC has become an immense threat to the public health system in China (3–5). Although the management for HCC has been greatly improved during the past few decades, the management option for advanced HCC patients is still insufficient (6, 7). Currently, treatment modalities of advanced HCC are limited, which include programmed cell death protein 1 (PD-1) inhibitor plus vascular endothelial growth factor inhibitor and several tyrosine kinase inhibitors (TKIs) (such as sorafenib, lenvatinib, and donafenib) (8, 9). Therefore, it is necessary and urgent to explore potential novel targets for the treatment of HCC.

Small ubiquitin-like modifier (SUMO)-specific peptidase 2 (SENP2) particularly degrades the conjunction between SUMO and proteins, thus participating in various cell functions (10). Besides, SENP2 is also regarded as an anti-oncogene in several cancers. For instance, it is proposed that SENP2 overexpression restores the sensitivity to doxorubicin in doxorubicin-resistant breast cancer cells and suppresses the nuclear factor-κB (NF-κB) pathway (11). Meanwhile, in bladder cancer cells, a previous study suggests that SENP2 overexpression inhibits the transforming growth factor-β (TGF-β) pathway and the subsequent epithelial–mesenchymal transition (EMT), thus suppressing cell invasion (12). Moreover, an interesting study discloses that overexpression of SENP2 suppresses the progression of gastric cancer through stabilizing the N-Myc downstream-regulated gene 2 (13). However, few studies have been performed to explore the effect of SENP2 on the progression of HCC. Only two previous studies propose that SENP2 reduces the proliferation of HCC cells through modulating β-catenin stability (14, 15), whereas whether SENP2 could affect stemness and sensitivity to targeted therapy in HCC remains unclear.

In the present study, SENP2 modification was conducted in HCC cell lines, followed by the detection of stemness, sorafenib sensitivity, and the downstream pathway; besides, a compensation experiment was subsequently performed to validate its molecule mechanism, aiming to provide a potential option for the treatment of HCC.

## Methods

### Cells and Reagents

The cells and reagents used for this study are as follow THLE-3 (ATCC, USA), Hep3B (ATCC, USA), Li7 (RCB, Japan), Huh7 (RCB, Japan); BEGM Bullet Kit (Lonza, Switzerland); Eagle’s Minimum Essential Medium (EMEM) (Hyclone, USA); RPMI-1640 medium (Hyclone, USA); Dulbecco’s Modified Eagle Medium (DMEM) (Hyclone, USA); fetal bovine serum (FBS) (Hyclone, USA); pcDNA-NC plasmid (Genepharma, China); pcDNA-SENP2 plasmid (Genepharma, China); pGPH1-NC plasmid (Genepharma, China); pGPH1-SENP2 plasmid (Genepharma, China); SC79 (MCE, China); pcDNA-CTNNB1 plasmid (Genepharma, China); transfection reagent (Sangon, China); Trizol (Beyotime, China); qPCR RT Kit (Toyobo, Japan); qPCR Master Mix (Toyobo, Japan); RIPA buffer (Beyotime, China); BCA kit (Beyotime, China); 4–20% precast gel (Willget, China); nitrocellulose membrane (PALL, USA); BSA (Beyotime, China); ECL kit (Beyotime, China); SENP2 antibody (1:2,000) (Invitrogen, USA); protein kinase B (AKT) antibody (1:1,000) (Invitrogen, USA); pAKT antibody (1:1,000) (Invitrogen, USA); glycogen synthase kinase-3β (GSK3β) antibody (1:1,000) (CST, USA); pGSK3β antibody (1:1,000) (CST, USA); β-catenin (CTNNB1) antibody (1:1,000) (Invitrogen, USA); Sox2 antibody (1:1,000) (CST; USA); Nanog antibody (1:1,000) (CST, USA); Oct4 (1:50,000) (Invitrogen, USA); pMEK1/2 antibody (1:1,000) (Invitrogen, USA); MEK1/2 antibody (1:2,000) (Invitrogen, USA); pERK1/2 antibody (1:1,000) (Invitrogen, USA); ERK1/2 antibody (1:2,000) (Invitrogen, USA); β-actin antibody (1:20,000); Goat Anti-Mouse IgG (H+L) HRP antibody (1:100,000) (Invitrogen, USA); cell counting kit-8 (Sangon, China); Dulbecco’s Modified Eagle Medium/Nutrient Mixture F-12 (DMEM/F12) (Gibco, USA); epidermal growth factor (EGF) (PeproTech, USA); basic fibroblast growth factor (bFGF) (PeproTech, USA); insulin (Sangon, China); Alexa Fluor 488-linked CD133 antibody (1:50) (eBioscience, USA); and Matrigel Basement Membrane Matrix (BD, USA).

### SENP2 Expression in HCC Tissues and Cells

A total of 10 paraffin sections of the HCC tumor and paired adjacent noncancerous tissues were obtained after being approved by the Ethics Committee of our institution. The expression of SENP2 in tissues was determined by immunohistochemistry (IHC) according to the methods described in the previous study (16).

THLE-3 cells were cultured with BEGM Bullet Kit; Hep3B cells were cultured with EMEM; Li7 and Huh7 cells were cultured with RPMI-1640 and DMEM, respectively. All the culture medium was supplemented with 10% FBS. The expression of SENP2 in cells was evaluated by reverse transcription-quantitative polymerase chain reaction (RT-qPCR) and Western blot.

### Cancer Stem Cell isolation and SENP2 Expression Evaluation

The Huh7 and Hep3B cells were collected and stained with Alexa Fluor 488-linked CD133 antibody on ice for 30 min. Afterward, the CD133-positive (CD133^+^) cells were collected with a flow cytometer (BD, USA) and defined as CSC. Meanwhile, the normally cultured Huh7 and Hep3B cells were served as normal control. The expression of SENP2 in CSC and normal control was assessed by RT-qPCR and Western blot.

### SENP2 Plasmid Transfection

The control and SENP2 overexpression (pcDNA-NC and pcDNA-SENP2) plasmids, control, and SENP2 knockdown (pGPH1-NC and pGPH1-SENP2) plasmids were transfected into Huh7 and Hep3B cells with the application of transfection reagent according to the manufacturer’s protocols. The cells were categorized as pcDNA-NC, pcDNA-SENP2, pGPH1-NC, and pGPH1-SENP2 groups, accordingly.

### Compensation Experiment

The plasmids were transfected into Huh7 and Hep3B cells in the presence of transfection reagent, and 4 μg/ml SC79 was added and cultured with cells (17). Details of transfection and culture condition were as follows: (a) Normal group, the cells were cultured normally; (b) pcDNA-NC group, the cells were transfected with pcDNA-NC plasmid; (c) pcDNA-SENP2 group, the cells were transfected with pcDNA-SENP2 plasmid; (d) SC79 group, the cells were transfected with pcDNA-NC plasmid and incubated with SC79; (e) pcDNA-SENP2 + SC79 group, the cells were transfected with pcDNA-SENP2 plasmid and incubated with SC79; (f) pcDNA-CTNNB1 group, the cells were transfected with pcDNA-CTNNB1 plasmid; (g) pcDNA-SENP2 + pcDNA-CTNNB1 group, the cells were transfected with pcDNA-SENP2 and pcDNA-CTNNB1 plasmids.

### RT-qPCR

At 48 h after transfection, transfected cells were collected. Then, the total RNA of cells (including CSC and non-transfected cells) was extracted with Trizol. Next, 1 μg of total RNA was transcribed into cDNA with the application of qPCR RT Kit. After that, quantification was conducted by qPCR Master Mix, and the thermal cycle was as follows: 95°C for 60 s, 1 cycle; 95°C for 15 s, 61°C for 20 s, 40 cycles. Last, the result was calculated with the 2^−ΔΔCt^ method with β-actin serving as the internal reference. The primer sequences (5’-> 3’) were listed as follows: SENP2 forward, TTGGAGCCTGGTGGTGATTG; SENP2 reverse, TGTTGAGGAATCTCGTGTGGTT; β-actin forward, GGCACCACACCTTCTACAATGA, β-actin reverse, GGATAGCACAGCCTGGATAGC.

### Western Blot

The cells were collected at 48 h after transfection. Thereafter, cells (including CSC and non-transfected cells) were lysed by RIPA buffer and the total protein was quantified by BCA kit. Then 20 μg of protein was separated by 4%–20% precast gel and transferred to nitrocellulose membrane. The membrane was then blocked with 5% BSA for 1.5 h at 37°C. After blocking, the membrane was incubated with diluted primary antibodies at 4°C overnight, then incubated with secondary antibody at 37°C for 2 h. Last, the protein bands were developed with an ECL kit.

### Transwell Assay

The transwell assay was carried out at 48 h post transfection. The transwell chamber (Corning, USA) was pre-coated with Matrigel Basement Membrane Matrix at 37°C for 1 h. Then, 6 × 10^4^ cells in 200 μl of FBS-free EMEM or RPMI1640 medium were added in the transwell chamber. The lower chamber was filled with 500 μl of EMEM or RPMI1640 medium containing 10% FBS. After incubation for 24 h at 37°C, the non-invasive cells were wiped out. Meanwhile, the invasive cells were fixed with 4% paraformaldehyde and stained with 0.1% crystal violet. Last, the images were captured by an inverted microscope (Nikon, Japan).

### Scratch Wound Assay

At 48 h after transfection, scratch wound assay was conducted. A wound was scratched after the cells reach 80% confluence. Then, the cells were cultivated at 37°C for 24 h. The photos were taken by an inverted microscope (Nikon, Japan) at 0 h and 24 h after the wound was scratched. The wound area was analyzed by Image‐Pro Plus 6.0 (Media Cybernetics, USA).

### Sorafenib Sensitivity

At 48 h after transfection, the cells were harvested and seeded into a 96-well plate. Then, sorafenib solutions with a concentration of 0, 0.5, 1, 2, 4, and 8 μM were added and incubated with cells for another 24 h or 72 h. Last, the cells were incubated for 2 h in 100 μl of EMEM or RPMI1640 medium mixing with 10 μl of cell counting kit-8 reagent. Subsequently, the optical density (OD) value at 450 nm was evaluated by a microplate reader (BioTek, USA). The relative cell viability was calculated with an OD value of 0 μM setting as 100%. The IC_50_ of cells was evaluated by prohibit regression analysis.

### Flow Cytometry

The proportion of CD133^+^ cells was evaluated at 48 h after transfection. In brief, the cells were collected and counted. Then 1 × 10^5^ cells in 100 μl of buffer were stained with Alexa Fluor 488-linked CD133 antibody on ice for 30 min. Finally, the cells were analyzed with a flow cytometer (BD, USA).

### Sphere Formation Assay

The sphere formation was carried out at 48 h post-transfection. The cells were harvested, counted, and re-suspended. Next, cells with a number of 1,000 were seeded into an ultra-low attachment 24-well plate and cultured for 10 days in a sphere formation medium. The sphere formation medium was DMEM/F12 containing 20 ng/ml EGF, 10 ng/ml bFGF, and 5 μg/ml insulin. After culture, the number of spheres with a diameter larger than 50 μm was counted with an inverted microscope (Nikon, Japan), and the diameter of the sphere was evaluated.

### Statistical Analysis

GraphPad Prism 7.02 (GraphPad Software Inc., USA) was applied to analyze data and plot graphs. All data in this study were expressed as mean ± standard deviation (SD). Unpaired *t*-test, one-way ANOVA, Dunnett’s multiple comparison, and Tukey’s multiple comparison were used to assess the difference between two groups or among groups. The prohibit regression analysis was performed by SPSS Software 23.0 (IBM, USA). The statistical significance was defined as *p* < 0.05.

## Results

### SENP2 Expression

**Figure 1:**
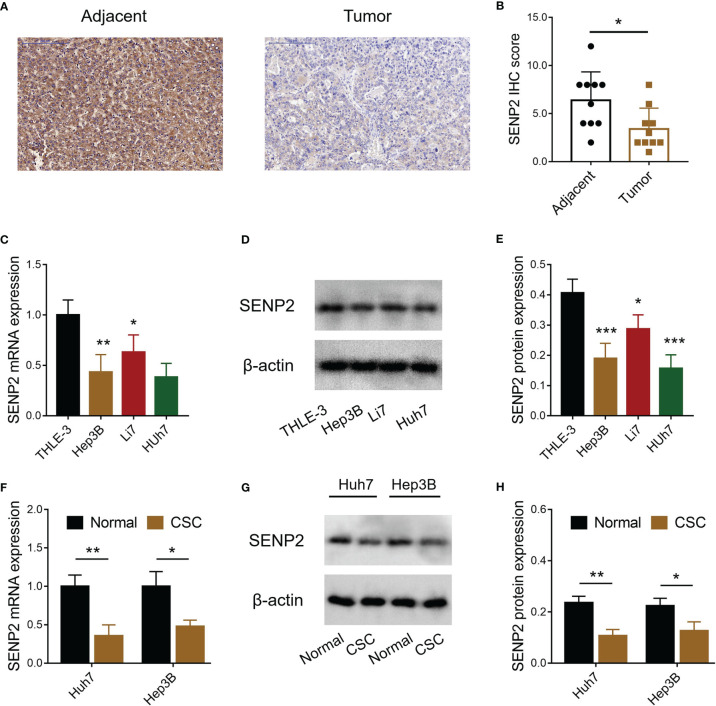
Detection of SENP2 expression. Representative images of SENP2 expression detection by IHC assay in HCC tumor and adjacent tissues **(A)**. Comparison of SENP2 IHC score between HCC tumor and adjacent tissues **(B)**. Comparison of SENP2 mRNA expression among the THLE-3 cell line and HCC cell lines **(C)**. Detection of SENP2 protein expression by Western blot in the THLE-3 cell line and HCC cell lines **(D)**. Comparison of SENP2 protein expression among the THLE-3 cell line and HCC cell lines **(E)**. Comparison of SENP2 mRNA expression between normal HCC cells and HCC CSCs **(F)**. Detection of SENP2 protein expression by Western blot in normal HCC cells and HCC CSCs **(G)**. Comparison of SENP2 protein expression between normal HCC cells and CSCs of HCC CSCs **(H)**. SENP2, Small ubiquitin-like modifier specific peptidase 2; IHC, immunohistochemical; HCC, hepatocellular carcinoma; CSC, cancer stem cell; *p < 0.05; **p < 0.01; ***p < 0.001.

SENP2 expression in HCC tumor tissues and adjacent tissues was detected by IHC (**Figure 1A**), which revealed that SENP2 IHC score was reduced in tumor tissues compared with adjacent tissues (*p* < 0.05) (**Figure 1B**). Meanwhile, the level of SENP2 was also decreased in HCC cell lines (including Hep3B, Li7, and Huh7) compared to the THLE-3 cell line (all *p* < 0.05) (**Figures 1C**–**E**). Since SENP2 was dramatically decreased in Hep3B and Huh7 cells, these two cells were selected for further experiments. Moreover, the CSCs of Huh7 and Hep3B cells were isolated for SENP2 expression detection, which revealed that the level of SENP2 was downregulated in CSCs of Huh7 and Hep3B cells compared with normal Huh7 and Hep3B cells, respectively (all *p* < 0.05) (**Figures 1F**–**H**).

### SENP2 Regulated Stemness and Sensitivity to Sorafenib in HCC Cell Lines

**Figure 2:**
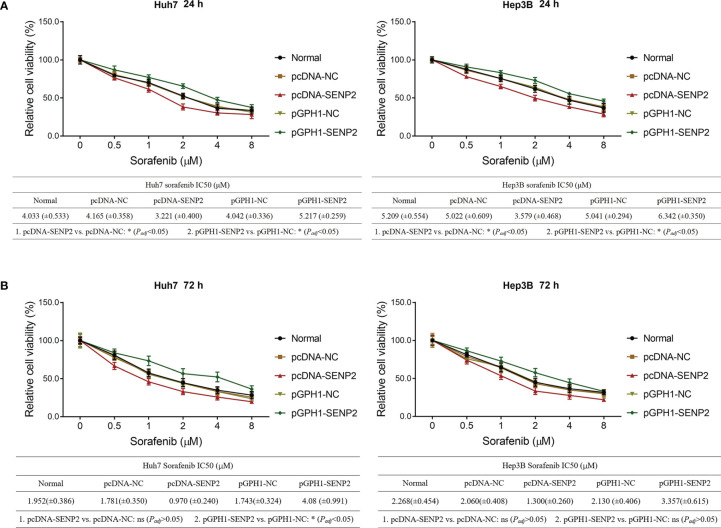
Sorafenib sensitivity after plasmid transfection. Relative cell viability under different doses of sorafenib treatment for 24 h and comparison of IC_50_ value of sorafenib treatment for 24 h among groups in Huh7 and Hep3B cells after transfection **(A)**. Relative cell viability under different doses of sorafenib treatment for 72 h and comparison of IC_50_ of sorafenib treatment for 72 h among groups in Huh7 and Hep3B cells after transfection **(B)**. SENP2, Small ubiquitin-like modifier specific peptidase 2; NC, negative control; IC_50_, half maximal inhibitory concentration.

**Figure 3:**
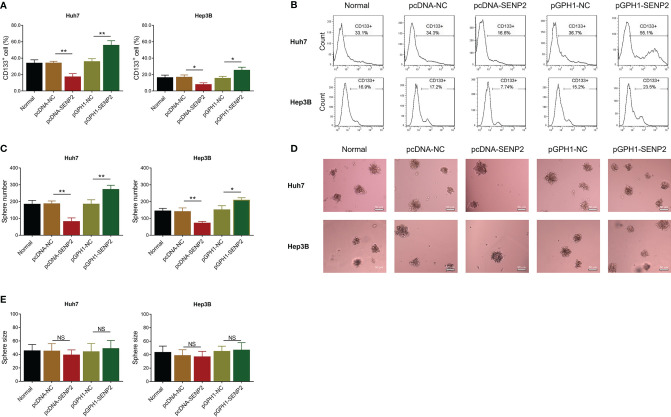
Stemness after plasmid transfection. Comparison of CD133^+^ cell proportion among groups in Huh7 and Hep3B cells after transfection **(A)**. Representative images of CD133^+^ cell proportion detection by flow cytometry assay **(B)**. Comparison of sphere number among groups in Huh7 and Hep3B cells after transfection **(C)**. Representative images of sphere formation assay **(D)**. Comparison of sphere size among groups in Huh7 and Hep3B cells after transfection **(E)**. SENP2, Small ubiquitin-like modifier specific peptidase 2; NC, negative control; CD133^+^, cluster of differentiation 133 positive; *p < 0.05; **p < 0.01; NS, not significant.

In order to explore the regulation of SENP2 on stemness, sorafenib sensitivity, and the downstream targets, plasmid transfection was conducted in Huh7 and Hep3B cells; the efficiency of plasmid transfection was assessed by RT-qPCR and Western blot (**Supplementary Figures 1A**–**C**). The subsequent CCK-8 assay revealed that SENP2 overexpression enhanced sensitivity to sorafenib, while SENP2 knockdown reduced that in Huh7 cells and Hep3B cells (all *p* < 0.05) (**Figures 2A, B**). Moreover, SENP2 overexpression reduced the proportion of CD133^+^ cells and sphere formation ability, while SENP2 knockdown showed the opposite effect in Huh7 and Hep3B cells (all *p* < 0.05), whereas SENP2 overexpression or knockdown showed little effect on sphere size (all *p* > 0.05) (**Figures 3A**–**E**). Besides, SENP2 overexpression also reduced the expression of stemness markers Sox2, Nanog, and Oct4, and decreased cell migration and invasion, but SENP2 knockdown presented the opposite effects in Huh7 and Hep3B cells (all *p* < 0.05) (**Supplementary Figures 2A**–**F**).

### SENP2 Inactivated AKT/GSK3β/CTNNB1 Signaling in HCC Cell Lines

**Figure 4:**
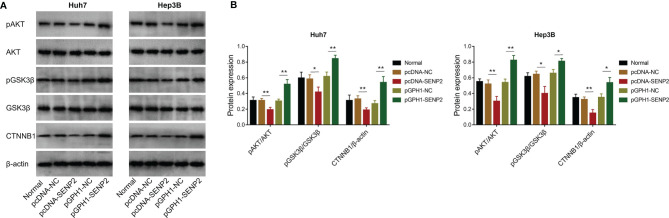
AKT/GSK3β/CTNNB1 pathway after plasmid transfection. Detection of protein expressions by Western blot in Huh7 and Hep3B cells after transfection **(A)**. Comparison of pAKT/AKT, pGSK3β/GSK3β, and CTNNB1 expressions among groups in Huh7 and Hep3B cells after transfection **(B)**. SENP2, Small ubiquitin-like modifier specific peptidase 2; NC, negative control; AKT, protein kinase B; GSK3β, glycogen synthase kinase-3β; CTNNB1, β-catenin; *p < 0.05; **p < 0.01.

The potential downstream signaling pathway of SENP2 was evaluated by Western blot, which revealed that SENP2 overexpression decreased the phosphorylation of AKT and GSK3β, as well as CTNNB1 expression in both Huh7 and Hep3B cells; in contrast, SENP2 knockdown increased the phosphorylation of AKT and GSK3β, as well as CTNNB1 expression in Huh7 and Hep3B cells (all *p* < 0.05) (**Figures 4A, B**). Besides, SENP2 overexpression promoted the phosphorylation of MEK and ERK, while SENP2 knockdown reduced that (all *p* < 0.05) (**Supplementary Figures 2A, B**).

### AKT/GSK3β/CTNNB1 Signaling Was Necessary for the Regulation of SENP2 on Stemness and Sorafenib Sensitivity in HCC Cell Lines

**Figure 5:**
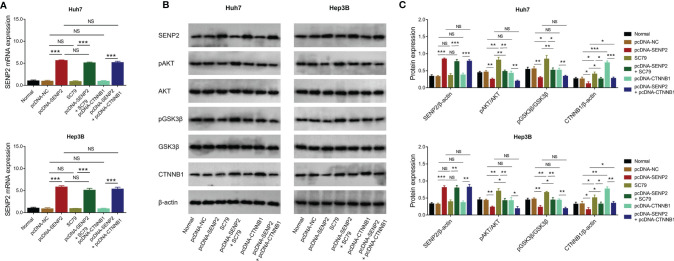
Protein and mRNA expressions in the compensation experiment. Comparison of SENP2 mRNA expression among groups in Huh7 and Hep3B cells in the compensation experiment **(A)**. Detection of protein expressions by Western blot in Huh7 and Hep3B cells in the compensation experiment **(B)**. Comparison of SENP2, pAKT/AKT, pGSK3β/GSK3β, and CTNNB1 expressions among groups in Huh7 and Hep3B cells in the compensation experiment **(C)**. SENP2, Small ubiquitin-like modifier specific peptidase 2; NC, negative control; AKT, protein kinase B; GSK3β, glycogen synthase kinase-3β; CTNNB1, β-catenin; *p < 0.05; **p < 0.01; ***p < 0.001; NS, not significant.

In order to clarify whether SENP2 regulated stemness and sensitivity to sorafenib through AKT/GSK3β/CTNNB1 signaling in HCC cell lines, compensation experiments were conducted. Data revealed that SC79 (activator of AKT) treatment or CTNNB1 overexpression did not affect SENP2 expression in Huh7 and Hep3B cells (all *p* > 0.05) (**Figures 5A**–**C**). Meanwhile, their regulation on the phosphorylation of AKT and GSK3β, as well as CTNNB1 expression was explored by Western blot (**Figures 5B, C**).

**Figure 6:**
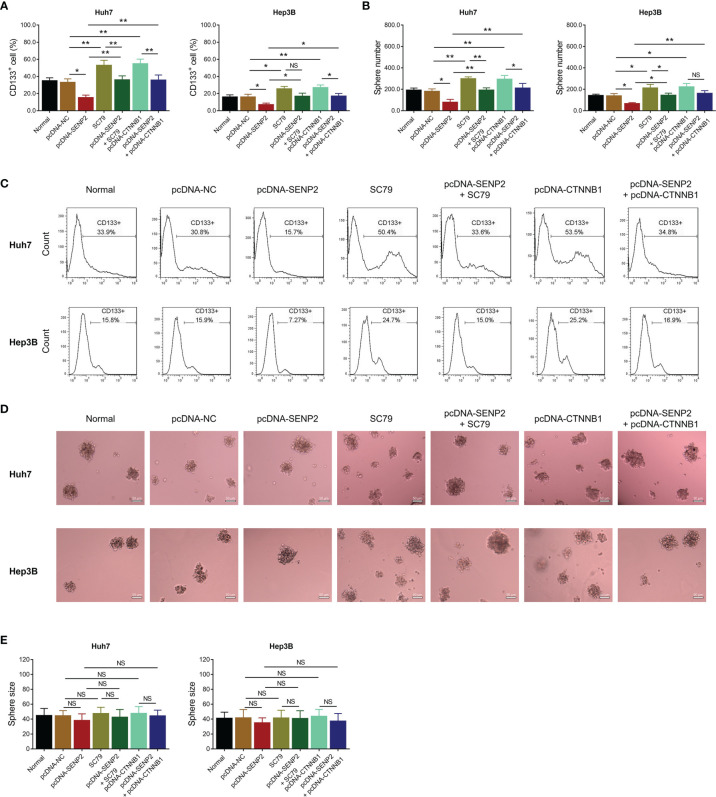
Stemness in the compensation experiment. Comparison of CD133^+^ cell proportion **(A)** and sphere number **(B)** among groups in Huh7 and Hep3B cells in the compensation experiment. Representative images of CD133^+^ cell proportion detection by flow cytometry assay **(C)** and sphere formation assay **(D)**. Comparison of sphere size among groups in Huh7 and Hep3B cells in the compensation experiment **(E)**. SENP2, Small ubiquitin-like modifier specific peptidase 2; NC, negative control; CTNNB1, β-catenin; CD133^+^, cluster of differentiation 133 positive; *p < 0.05; **p < 0.01; NS, not significant.

Further experiments disclosed that SC79 treatment or CTNNB1 overexpression increased the proportion of CD133^+^ cells and sphere formation ability in Huh7 and Hep3B cells (all *p* < 0.05) (**Figures 6A**–**D**). Meanwhile, SC79 treatment or CTNNB1 overexpression compensated SENP2 overexpression-induced decrease of CD133^+^ cell proportion and sphere formation ability in Huh7 and Hep3B cells (all *p* < 0.05), whereas sphere size was hardly affected by SC79 treatment or CTNNB1 overexpression (all *p* > 0.05) (**Figures 6A**–**E**).

**Figure 7:**
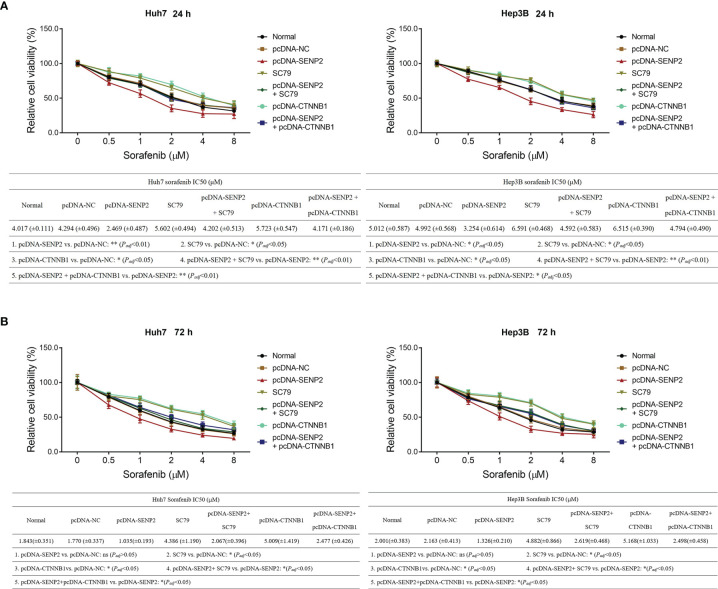
Sorafenib sensitivity in compensation experiment. Relative cell viability under different doses of sorafenib treatment for 24 h and comparison of IC_50_ value of sorafenib treatment for 24 h among groups in Huh7 and Hep3B cells in the compensation experiment **(A)**. Relative cell viability under different doses of sorafenib treatment for 72 h and comparison of IC_50_ value of sorafenib treatment for 72 h among groups in Huh7 and Hep3B cells in the compensation experiment **(B)**. SENP2, Small ubiquitin-like modifier specific peptidase 2; NC, negative control; CTNNB1, β-catenin; IC_50_, half maximal inhibitory concentration.

Moreover, the CCK-8 assay revealed that SC79 treatment or CTNNB1 overexpression not only reduced sensitivity to sorafenib, but also weakened SENP2 overexpression-induced enhancement of sorafenib sensitivity in Huh7 and Hep3B cells (all *p* < 0.05) (**Figures 7A, B**).

## Discussion

SUMOylation is a process of post-translational modification of a protein by reversible conjunction of SUMO with selected proteins, which is involved in various biological processes, including cancer progression (18, 19). For instance, the SUMOylation of flotillin-1 at lysine-51 and lysine-195 in prostate cancer cells enhances the stabilization of Snail and induces the upregulation of EMT-related genes, thus enhancing the metastatic potential of prostate cancer (20). Another interesting study suggests that SUMOylation of AKT at lysine-276 activates AKT, subsequently promoting cell proliferation and migration in non-small cell lung cancer (21). Meanwhile, in breast cancer cells, SUMOylation of monocarboxylate transporter 4 at lysine-448 is critical for cell proliferation (22). In HCC, it is also supposed that SUMOylation plays an essential role in cancer progression. For example, the SUMOylation of exportin-5 at lysine-125 promotes the proliferation, migration, and invasion of HCC cell lines (23); Shp2, a key regulation of Ras/extracellular signal-regulated kinase pathway, is SUMOylated at lysine-590, thus facilitating xenografted tumor growth (24); and suppressing of phosphoenolpyruvate carboxykinase SUMOylation inhibits HCC xenografted tumor progression (25). Therefore, it is reasonable to hypothesize that SENP2, as an enzyme, inhibits SUMOylation and suppresses the progression of several cancers, including HCC. However, few studies investigate this issue. In the present study, it was revealed that SENP2 was insufficiently expressed both in HCC tissues than in adjacent non-cancerous tissues, and in HCC cell lines compared with normal liver epithelial cells. Combining with previous studies, it could be deduced that SENP2 was a tumor suppressor in various cancers; however, studies should be further conducted to verify this. Besides, since the staining of the whole tissue sample might include other cells apart from HCC cells, it might be necessary to conduct dual staining of HCC markers (such as alpha-fetoprotein) combined with SENP2 to confirm the level of SENP2 in HCC tissues. Moreover, the present study also disclosed that SENP2 was lower in CSCs of HCC cells compared to normal HCC cells. According to previous studies, SUMOylation is a key regulator of HCC CSCs (26). Therefore, as an inhibitor of SUMOylation, SENP2 was decreased in CSCs of HCC cells.

Cancer stemness refers to the stem-cell-like phenotype of the cancer cells (27). It is widely accepted that stemness is critical for cancer metastasis and recurrence and contributes to drug resistance, thus directly resulting in a worse prognosis in patients with cancer (28). In HCC, several factors have been identified that are highly relevant to stemness, including p53 mutation, β-catenin mutation, and telomerase reverse transcriptase promoter mutation (28). Recently, it is also proposed that SUMOylation plays a key role in HCC stemness (29), which highlights the possibility of SENP2 as a regulator for HCC stemness. Meanwhile, as mentioned earlier, our study disclosed that SENP2 was decreased in CSCs of HCC cells. Therefore, the effect of SENP2 on the stemness of HCC cells was investigated. Following the plasmid transfection, the flow cytometry, sphere formation, and scratch and invasion assays, combined with detection of stemness markers by Western blot revealed that the overexpression of SENP2 reduced, while the knockdown of SENP2 promoted the stemness of HCC cell lines. Previous studies indicate that SENP2 reduces the stability of β-catenin in HCC, which is a key contributor to HCC stemness (14, 15). Subsequently, since stemness is a vital contributor to drug resistance, the effect of SENP2 on sensitivity to sorafenib was also investigated since sorafenib is generally recommended for the treatment of advanced HCC (8, 9). Data revealed that the overexpression of SENP2 increased the sensitivity to sorafenib, while the knockdown of SENP2 showed an opposite effect. These findings highlighted the potential of SENP2 as a therapeutic option for HCC. However, further experiments were needed to verify the effect of SENP2 on sorafenib sensitivity in HCC-bearing mice. Besides, identification of stemness using CD133 combined with CD44 or EpCAM could be conducted further.

The AKT/GSK3β/CTNNB1 signaling pathway is critical for the progression of several cancers, including HCC. AKT is a serine threonine kinase activated by phosphoinositide 3-kinase (30). Once phosphorylated, AKT translocates to various sublocations of the cells and phosphorylates its downstream targets, including GSK3β (30). Subsequently, pGSK3β activates CTNNB1, which further promotes proliferation, migration, invasion, and triggers drug resistance in HCC cells (31). Besides, the AKT/GSK3β pathway is also highly involved in the regulation of sorafenib sensitivity in HCC (32–34). More importantly, it is proposed that SUMOylation participates in the activation of AKT and CTNNB1 (21, 35). Therefore, it could be deduced that SENP2 might regulate the stemness of HCC cells through the AKT/GSK3β/CTNNB1 signaling pathway. Although previous studies have revealed the regulation of SENP2 on AKT and CTNNB1 (14, 36), whether it could exert similar effects in HCC cell lines remained unclear. The present study disclosed that the overexpression of SENP2 inhibited the AKT/GSK3β/CTNNB1 signaling pathway, while the knockdown of SENP2 presented a reverse effect. Furthermore, in the present study, the compensation experiments disclosed that activating AKT or overexpression of CTNNB1 promoted stemness, reduced sorafenib sensitivity, and further attenuated the effect of SENP2 overexpression on stemness and sorafenib sensitivity in HCC cells, indicating that the AKT/GSK3β/CTNNB1 signaling pathway was essential for the regulation of SENP2 in stemness and sensitivity to sorafenib in HCC cell lines. Moreover, SUMOylation is also reported to regulate pathways that associate with proliferation, migration, and invasion, including the MEK/ERK pathway (37, 38). In the present study, it was found that SENP2, an inhibitor of SUMOylation, also suppressed the MEK/ERK pathway in HCC cell lines. Besides, evaluating stemness markers, p-AKT, and β-catenin in the HCC tissues might further verify our findings.

To be conclusive, SENP2 suppresses HCC stemness and promotes its sensitivity to sorafenib through inactivating the AKT/GSK3β/CTNNB1 signaling pathway, indicating that SENP2 might be a potential treatment option for the management of HCC.

## Data Availability Statement

The original contributions presented in the study are included in the article/**Supplementary Material**. Further inquiries can be directed to the corresponding author.

## Author Contributions

XT and ZL made substantial contributions to the design of the present study. Data acquisition was performed by XT, BL, CZ, WT, SL, and YX. Data analysis was performed by RD, and data interpretation was performed by XT, BL, CZ, WT, SL, and ZL. XT and ZL critically revised the manuscript for important intellectual content. All authors approved the final version of the manuscript. All authors agree to be accountable for all aspects of the work in ensuring that questions related to the accuracy or integrity of the work are appropriately investigated and resolved.

## Funding

This study was supported by the National Natural Science Foundation of China (No. 81974458) and the China Hunan Provincial Science/Technology Department (2019RS1042, 2019TP1035, and 2018RS3072).

## Conflict of Interest

The authors declare that the research was conducted in the absence of any commercial or financial relationships that could be construed as a potential conflict of interest.

## Publisher’s Note

All claims expressed in this article are solely those of the authors and do not necessarily represent those of their affiliated organizations, or those of the publisher, the editors and the reviewers. Any product that may be evaluated in this article, or claim that may be made by its manufacturer, is not guaranteed or endorsed by the publisher.

## Supplementary Material

Supplementary Figure 1 SENP2 expression after plasmid transfection. Comparison of SENP2 mRNA expression among groups in Huh7 and Hep3B cells after transfection **(A)**; Detection of SENP2 protein expression by western blot in Huh7 and Hep3B cells after transfection **(B)**; Comparison of SENP2 protein expression among groups in Huh7 and Hep3B cells after transfection **(C)**. SENP2: Small ubiquitin-like modifier specific peptidase 2; NC: negative control; **: *p*<0.01; ***: *p*<0.001.

Supplementary Figure 2 Stemness markers, cell migration and invasion after plasmid transfection. Representative images of stemness markers and MEK/ERK pathway detection by western blot in Huh7 and Hep3B cells after transfection **(A)**; Comparison of Sox2, Nanog, Oct4, pMEK/MEK and pERK/ERK among groups in Huh7 and Hep3B cells after transfection **(B)**; Representative images of scratch assay **(C)** and comparison of migration rate **(D)** among groups in Huh7 and Hep3B cells after transfection; Representative images of invasion assay **(E)** and comparison of invasive cell count **(F)** among groups in Huh7 and Hep3B cells after transfection. SENP2: Small ubiquitin-like modifier specific peptidase 2; ERK: extracellular signal-regulated kinases; MEK: mitogen-activated protein kinase/ERK kinase; NC: negative control; *: *p*<0.05; **: *p*<0.01.

## References

[1] McGlynn KA Petrick JL El-Serag HB. Epidemiology of Hepatocellular Carcinoma. Hepatology (2021) 73 Suppl:14–3. doi: 10.1002/hep.31288 PMC7577946 32319693

[2] Singal AG Lampertico P Nahon P. Epidemiology and Surveillance for Hepatocellular Carcinoma: New Trends. J Hepatol (2020) 72(2):250–61. doi: 10.1016/j.jhep.2019.08.025 PMC6986771 31954490

[3] Wang FS Fan JG Zhang Z Gao B Wang HY. The Global Burden of Liver Disease: The Major Impact of China. Hepatology (2014) 60(6):2099–108. doi: 10.1002/hep.27406 PMC4867229 25164003

[4] Xiao J Wang F Wong NK Lv Y Liu Y Zhong J. Epidemiological Realities of Alcoholic Liver Disease: Global Burden, Research Trends, and Therapeutic Promise. Gene Expr (2020) 20(2):105–18. doi: 10.3727/105221620X15952664091823 PMC7650014 32690129

[5] Yang JD Hainaut P Gores GJ Amadou A Plymoth A Roberts LR. A Global View of Hepatocellular Carcinoma: Trends, Risk, Prevention and Management. Nat Rev Gastroenterol Hepatol (2019) 16(10):589–604. doi: 10.1038/s41575-019-0186-y 31439937 PMC6813818

[6] Raees A Kamran M Ozkan H Jafri W. Updates on the Diagnosis and Management of Hepatocellular Carcinoma. Euroasian J Hepatogastroenterol (2021) 11(1):32–40. doi: 10.5005/jp-journals-10018-1335 34316462 PMC8286363

[7] Foerster F Galle PR. The Current Landscape of Clinical Trials for Systemic Treatment of HCC. Cancers (Basel) (2021) 13(8):1962. doi: 10.3390/cancers13081962 PMC8073471 33921731

[8] Gordan JD Kennedy EB Abou-Alfa GK Beg MS Brower ST Gade TP. Systemic Therapy for Advanced Hepatocellular Carcinoma: ASCO Guideline. J Clin Oncol (2020) 38(36):4317–45. doi: 10.1200/JCO.20.02672 33197225

[9] Chen LT Martinelli E Cheng AL Pentheroudakis G Qin S Bhattacharyya GS. Pan-Asian Adapted ESMO Clinical Practice Guidelines for the Management of Patients With Intermediate and Advanced/Relapsed Hepatocellular Carcinoma: A TOS-ESMO Initiative Endorsed by CSCO, ISMPO, JSMO, KSMO, MOS and SSO. Ann Oncol (2020) 31(3):334–51. doi: 10.1016/j.annonc.2019.12.001 32067677

[10] Heo KS. Regulation of Post-Translational Modification in Breast Cancer Treatment. BMB Rep (2019) 52(2):113–8. doi: 10.5483/BMBRep.2019.52.2.017 PMC6443327 30638182

[11] Gao X Wu Y Qiao L Feng X. SENP2 Suppresses NF-kappaB Activation and Sensitizes Breast Cancer Cells to Doxorubicin. Eur J Pharmacol (2019) 854:179–86. doi: 10.1016/j.ejphar.2019.03.051 30940449

[12] Tan M Zhang D Zhang E Xu D Liu Z Qiu J. SENP2 Suppresses Epithelial-Mesenchymal Transition of Bladder Cancer Cells Through Desumoylation of TGF-betaRI. Mol Carcinog (2017) 56(10):2332–41. doi: 10.1002/mc.22687 28574613

[13] Hu XY Liu Z Zhang KL Feng J Liu XF Wang LY. SUMO-Specific Protease 2-Mediated Desumoylation is Required for NDRG2 Stabilization in Gastric Cancer Cells. Cancer biomark (2017) 21(1):195–201. doi: 10.3233/CBM-170651 29060933 PMC13075757

[14] Shen HJ Zhu HY Yang C Ji F. SENP2 Regulates Hepatocellular Carcinoma Cell Growth by Modulating the Stability of Beta-Catenin. Asian Pac J Cancer Prev (2012) 13(8):3583–7. doi: 10.7314/apjcp.2012.13.8.3583 23098437

[15] Jiang QF Tian YW Shen Q Xue HZ Li K. SENP2 Regulated the Stability of Beta-Catenin Through WWOX in Hepatocellular Carcinoma Cell. Tumour Biol (2014) 35(10):9677–82. doi: 10.1007/s13277-014-2239-8 24969559

[16] Cui Y Wu X Lin C Zhang X Ye L Ren L. AKIP1 Promotes Early Recurrence of Hepatocellular Carcinoma Through Activating the Wnt/beta-Catenin/CBP Signaling Pathway. Oncogene (2019) 38(27):5516–29. doi: 10.1038/s41388-019-0807-5 30936461

[17] Bi J Liu Q Sun Y Hu X He X Xu C. CXCL14 Inhibits the Growth and Promotes Apoptosis of Hepatocellular Carcinoma Cells via Suppressing Akt/mTOR Pathway. J Recept Signal Transduct Res (2020) 41(6):593–603. doi: 10.1080/10799893.2020.1837870 33108937

[18] Eifler K Vertegaal ACO. SUMOylation-Mediated Regulation of Cell Cycle Progression and Cancer. Trends Biochem Sci (2015) 40(12):779–93. doi: 10.1016/j.tibs.2015.09.006 PMC4874464 26601932

[19] Tomasi ML Ramani K. SUMOylation and Phosphorylation Cross-Talk in Hepatocellular Carcinoma. Transl Gastroenterol Hepatol (2018) 3:20. doi: 10.21037/tgh.2018.04.04 29780898 PMC5945704

[20] Jang D Kwon H Choi M Lee J Pak Y. Sumoylation of Flotillin-1 Promotes EMT in Metastatic Prostate Cancer by Suppressing Snail Degradation. Oncogene (2019) 38(17):3248–60. doi: 10.1038/s41388-018-0641-1 PMC6756018 30631151

[21] Li R Wei J Jiang C Liu D Deng L Zhang K. Akt SUMOylation Regulates Cell Proliferation and Tumorigenesis. Cancer Res (2013) 73(18):5742–53. doi: 10.1158/0008-5472.CAN-13-0538 23884910

[22] Hu X Liu Z Duan X Han X Yuan M Liu L. Blocking MCT4 SUMOylation Inhibits the Growth of Breast Cancer Cells. Mol Carcinog (2021) 60(10):7028–14. doi: 10.1002/mc.23336 34347919

[23] Lin D Fu Z Yang G Gao D Wang T Liu Z. Exportin-5 SUMOylation Promotes Hepatocellular Carcinoma Progression. Exp Cell Res (2020) 395(2):112219. doi: 10.1016/j.yexcr.2020.112219 32763246

[24] Deng R Zhao X Qu Y Chen C Zhu C Zhang H. Shp2 SUMOylation Promotes ERK Activation and Hepatocellular Carcinoma Development. Oncotarget (2015) 6(11):9355–69. doi: 10.18632/oncotarget.3323 PMC4496222 25823821

[25] Bian XL Chen HZ Yang PB Li YP Zhang FN Zhang JY. Nur77 Suppresses Hepatocellular Carcinoma *via* Switching Glucose Metabolism Toward Gluconeogenesis Through Attenuating Phosphoenolpyruvate Carboxykinase Sumoylation. Nat Commun (2017) 8:14420. doi: 10.1038/ncomms14420 28240261 PMC5333363

[26] Shen Y Li Y Ma X Wan Q Jiang Z Liu Y. Connexin 43 SUMOylation Improves Gap Junction Functions Between Liver Cancer Stem Cells and Enhances Their Sensitivity to HSVtk/GCV. Int J Oncol (2018) 52(3):872–80. doi: 10.3892/ijo.2018.4263 29393359

[27] Prasad S Ramachandran S Gupta N Kaushik I Srivastava SK. Cancer Cells Stemness: A Doorstep to Targeted Therapy. Biochim Biophys Acta Mol Basis Dis (2020) 1866(4):165424. doi: 10.1016/j.bbadis.2019.02.019 30818002

[28] Tsui YM Chan LK Ng IO. Cancer Stemness in Hepatocellular Carcinoma: Mechanisms and Translational Potential. Br J Cancer (2020) 122(10):1428–40. doi: 10.1038/s41416-020-0823-9 PMC7217836 32231294

[29] Jiang Z Zhang C Liu X Ma X Bian X Xiao X. Dexamethasone Inhibits Stemness Maintenance and Enhances Chemosensitivity of Hepatocellular Carcinoma Stem Cells by Inducing Desumoylation of HIF1alpha and Oct4. Int J Oncol (2020) 57(3):780–90. doi: 10.3892/ijo.2020.5097 PMC7384854 32705164

[30] Dimri M Satyanarayana A. Molecular Signaling Pathways and Therapeutic Targets in Hepatocellular Carcinoma. Cancers (Basel) (2020) 12(2):491. doi: 10.3390/cancers12020491 PMC7072513 32093152

[31] Cervello M Augello G Cusimano A Emma MR Balasus D Azzolina A. Pivotal Roles of Glycogen Synthase-3 in Hepatocellular Carcinoma. Adv Biol Regul. (2017) 65:59–76 doi: 10.1016/j.jbior.2017.06.002 28619606

[32] Leung HW Leung CON Lau EY Chung KPS Mok EH Lei MML. EPHB2 Activates Beta-Catenin to Enhance Cancer Stem Cell Properties and Drive Sorafenib Resistance in Hepatocellular Carcinoma. Cancer Res (2021) 81(12):3229–40. doi: 10.1158/0008-5472.CAN-21-0184 33903122

[33] Xu S Ling S Shan Q Ye Q Zhan Q Jiang G. Self-Activated Cascade-Responsive Sorafenib and USP22 shRNA Co-Delivery System for Synergetic Hepatocellular Carcinoma Therapy. Adv Sci (Weinh) (2021) 8(5):2003042. doi: 10.1002/advs.202003042 33717848 PMC7927615

[34] Zhu YJ Zheng B Wang HY Chen L. New Knowledge of the Mechanisms of Sorafenib Resistance in Liver Cancer. Acta Pharmacol Sin (2017) 38(5):614–22. doi: 10.1038/aps.2017.5 PMC5457690 28344323

[35] Huang HJ Zhou LL Fu WJ Zhang CY Jiang H Du J. Beta-Catenin SUMOylation is Involved in the Dysregulated Proliferation of Myeloma Cells. Am J Cancer Res (2015) 5(1):309–20. PMC4300696 25628940

[36] Chen Y Xu T Li M Li C Ma Y Chen G. Inhibition of SENP2-Mediated Akt Desumoylation Promotes Cardiac Regeneration *via* Activating Akt Pathway. Clin Sci (Lond) (2021) 135(6):811–28. doi: 10.1042/CS20201408 33687053

[37] Kubota Y O'Grady P Saito H Takekawa M. Oncogenic Ras Abrogates MEK SUMOylation That Suppresses the ERK Pathway and Cell Transformation. Nat Cell Biol (2011) 13(3):282–91. doi: 10.1038/ncb2169 21336309

[38] Qu Y Chen Q Lai X Zhu C Chen C Zhao X. SUMOylation of Grb2 Enhances the ERK Activity by Increasing its Binding With Sos1. Mol Cancer (2014) 13:95. doi: 10.1186/1476-4598-13-95 24775912 PMC4021559

