# Supplementary figures and images for: SENP2 Reduces Hepatocellular Carcinoma Stemness and Improves Sorafenib Sensitivity Through Inactivating the AKT/GSK3β/CTNNB1 Pathway
Source: Front Oncol. 2021 Dec 7;11:773045. doi: 10.3389/fonc.2021.773045 (PMC8688154; doi:10.3389/fonc.2021.773045)

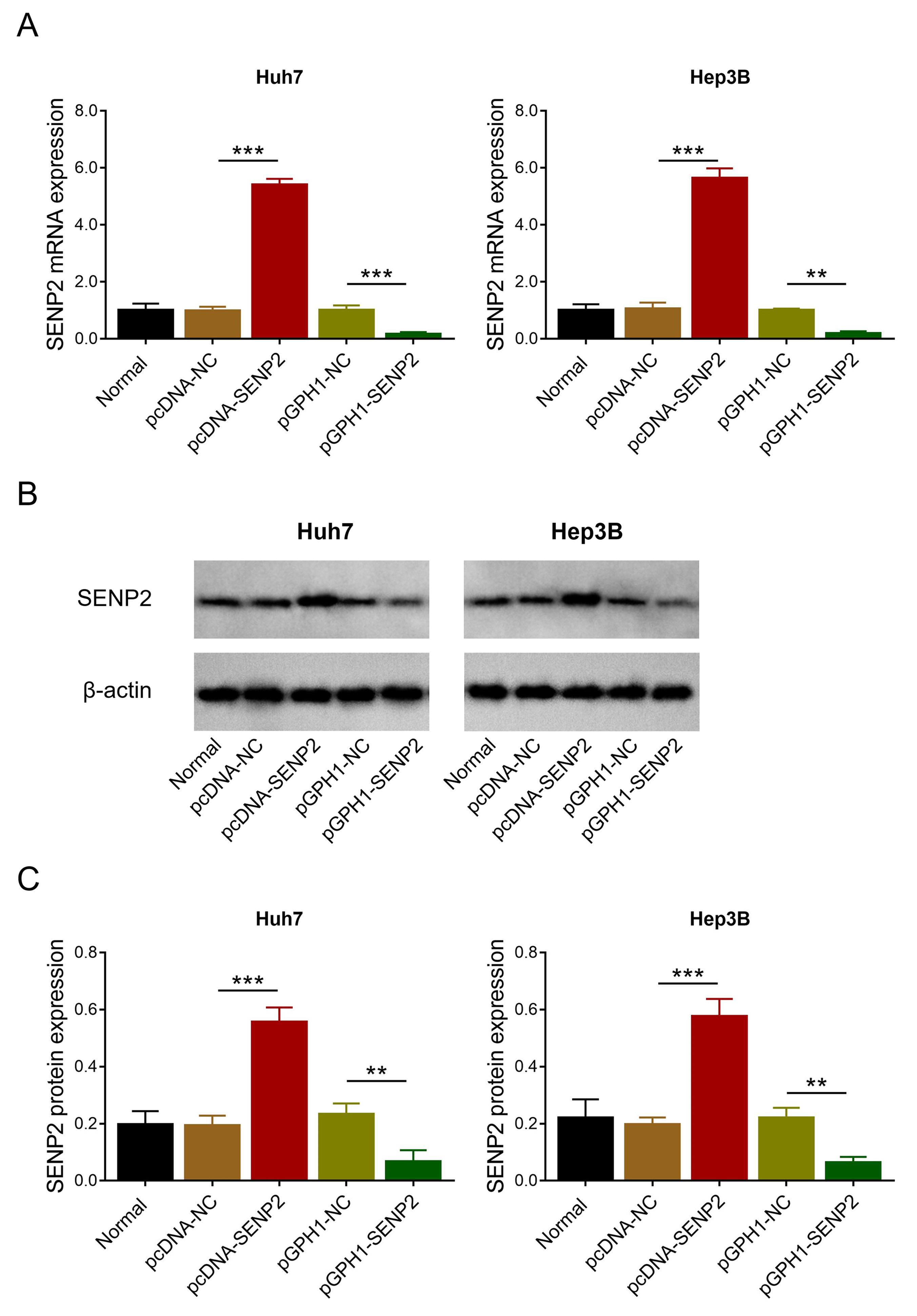

Supplement: Supplementary Figure 1 — SENP2 expression after plasmid transfection. Comparison of SENP2 mRNA expression among groups in Huh7 and Hep3B cells after transfection (A); Detection of SENP2 protein expression by western blot in Huh7 and Hep3B cells after transfection (B); Comparison of SENP2 protein expression among groups in Huh7 and Hep3B cells after transfection (C). SENP2: Small ubiquitin-like modifier specific peptidase 2; NC: negative control; **: p<0.01; ***: p<0.001. [file Image_1.tif]

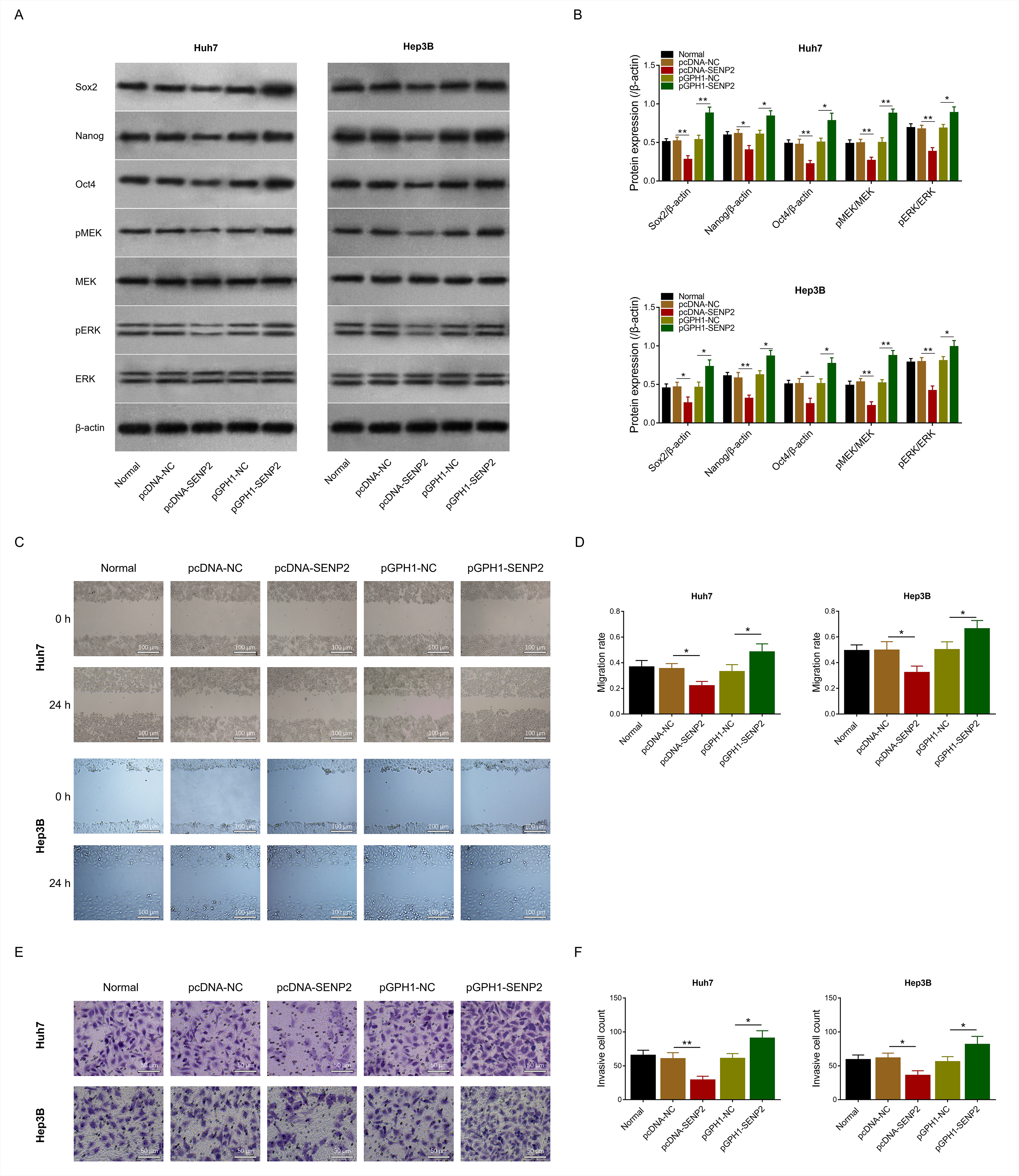

Supplement: Supplementary Figure 2 — Stemness markers, cell migration and invasion after plasmid transfection. Representative images of stemness markers and MEK/ERK pathway detection by western blot in Huh7 and Hep3B cells after transfection (A); Comparison of Sox2, Nanog, Oct4, pMEK/MEK and pERK/ERK among groups in Huh7 and Hep3B cells after transfection (B); Representative images of scratch assay (C) and comparison of migration rate (D) among groups in Huh7 and Hep3B cells after transfection; Representative images of invasion assay (E) and comparison of invasive cell count (F) among groups in Huh7 and Hep3B cells after transfection. SENP2: Small ubiquitin-like modifier specific peptidase 2; ERK: extracellular signal-regulated kinases; MEK: mitogen-activated protein kinase/ERK kinase; NC: negative control; *: p<0.05; **: p<0.01. [file Image_2.tif]
